# Mucormicosis: un dulce enemigo, serie de casos

**DOI:** 10.7705/biomedica.7120

**Published:** 2024-05-30

**Authors:** Santiago Manrique-Castaño, Luis Armando Velásquez-Trujillo, Mariana Ángel- Correa, José Humberto Bravo, Lorena Matta-Cortés

**Affiliations:** 1 Departamento de Medicina Interna, Escuela de Medicina, Facultad de Salud, Universidad del Valle, Cali, Colombia Universidad del Valle Departamento de Medicina Interna Escuela de Medicina, Facultad de Salud Universidad del Valle Cali Colombia; 2 Programa de Medicina y Cirugía, Escuela de Medicina, Facultad de Salud, Universidad del Valle, Cali, Colombia Universidad del Valle Programa de Medicina y Cirugía Escuela de Medicina, Facultad de Salud Universidad del Valle Cali Colombia; 3 Departamento de Patología, Facultad de Medicina, Pontificia Universidad Javeriana, Cali, Colombia Pontificia Universidad Javeriana Departamento de Patología Facultad de Medicina Pontificia Universidad Javeriana Cali Colombia

**Keywords:** mucormicosis, diabetes mellitus, huésped inmunocomprometido, microbiología, micología, mucormycosis, diabetes mellitus, immunocompromised host, microbiology, mycology

## Abstract

La mucormicosis es una infección fúngica poco frecuente causada por hongos del orden Mucorales, la cual se presenta en individuos inmunocomprometidos o con pérdida de la integridad de la barrera de piel o mucosas.

Se reportan cuatro casos de mucormicosis rinocerebral atendidos en un hospital de tercer nivel de Cali (Colombia) durante un periodo de tres años. Los cuatro pacientes presentaron diferentes cuadros clínicos y tiempos de evolución. Todos tenían diagnóstico de diabetes mellitus de tipo 2, de *novo* o previo, con una hemoglobina glucosilada de ingreso mayor del 10 % y en todos se descartaron otras enfermedades que explicaran su compromiso inmunitario. La mucormicosis se diagnosticó por la visualización directa de hifas hialinas sincitiales (*coenocytic*) en las biopsias tomadas.

El pilar del tratamiento fue la anfotericina B liposómica junto con el desbridamiento quirúrgico. Dos pacientes presentaron coinfección bacteriana. De los cuatro, uno firmó su egreso voluntario sin completar el tratamiento y otro falleció. Los dos pacientes restantes han asistido a los controles y han mostrado una adecuada evolución.

## Abstract (en)

Mucormycosis is a rare fungal infection caused by fungi of the Mucorales order that occurs in immunocompromised individuals or with loss of skin or mucosa barrier integrity.

This report presents four cases of rhinocerebral mucormycosis attended at a third-level hospital in Cali (Colombia) during a period of three years. All patients had different case histories and times of evolution. All four had a previous or *de novo* diagnosis of type 2 diabetes mellitus, with glycated hemoglobin higher than 10% on admission. We ruled out other possible pathologies that could explain their immunocompromised condition. Mucormycosis diagnosis was made with direct visualization of hyaline coenocytic hyphae on biopsies.

The basis of treatment was liposomal amphotericin B and surgical debridement. Two patients presented bacterial coinfection. One asked for voluntary discharge without having completed the treatment, and another one died. The remaining two have attended controls and had an adequate evolution.

La mucormicosis (antiguamente zigomicosis) es una infección causada por hongos filamentosos del orden Mucorales que puede causar infecciones potencialmente mortales en individuos inmunosuprimidos ^1^. Estos hongos son ubicuos y predominan en la materia orgánica en descomposición, producen hifas largas en forma de cintas con diámetros irregulares que, ocasionalmente, tienen tabiques. Las esporas que liberan tienen un diámetro de 3 a 11 pm y pueden introducirse en piel lesionada o inhalarse; este último es el mecanismo más frecuente de infección ^2^.

El agente patógeno más frecuente es *Rhizopus* spp. (47 %), seguido por *Mucor* spp. (18 %) y otros como *Lichtheimia* spp., *Rhizomucor* spp., *Cunninghamella* spp., *Apophysomyces* spp. y *Saksenaea* spp. ^3^. Los principales factores de riesgo para contraer mucormicosis son la pérdida de la integridad de la piel o las mucosas y el compromiso de la inmunidad celular. Las principales alteraciones asociadas reportadas son la diabetes mellitus mal controlada, las neoplasias hematológicas malignas, el trasplante de células madre hematopoyéticas, el uso de inmunosupresores y la terapia con deferoxamina ^1^^,^^3^.

La infección puede ser de localización rino-órbito-cerebral (22 %), pulmonar (14 %), cutánea (6 %), como también, sino-orbitaria, pulmonar, digestiva o diseminada ^4^.

El tratamiento debe ser multidisciplinario y se basa en la intervención quirúrgica con desbridamiento y limpieza de los bordes quirúrgicos, y la administración de un antifúngico. Respecto a este último, se ha encontrado actividad *in vitro* con anfotericina B, isavuconazol y posaconazol. Se han descrito diferentes combinaciones y el uso de equinocandinas en casos resistentes ^2^^,^^3^. La mortalidad varía entre el 40 y el 80 %, dependiendo de la extensión anatómica, el estado de inmunosupresión y el inicio oportuno del tratamiento médico y quirúrgico ^3^.

Los reportes en Colombia son pocos e informan una frecuencia de 0,2 por 100.000 habitantes. El compromiso mayor es de tipo rino-sino-órbito-cerebral. Sin embargo, un 27 % de los casos fueron reportados como infección de piel y tejidos blandos luego de erupciones volcánicas ^4^^-^^6^.

En esta serie, se reportan cuatro casos de mucormicosis en pacientes, al menos, con un factor inmunosupresor, variabilidad clínica y compromiso anatómico. El tratamiento antifúngico difirió en la mayoría de los casos, pero tuvo como base la anfotericina B. En el cuadro 1, se muestran las características, similitudes y diferencias que presentó cada paciente.

**Cuadro 1:** Características clínicas, tratamiento y resultado de los cuatro pacientes

Características	Paciente 1	Paciente 2	Paciente 3	Paciente 4
Sexo	Masculino	Masculino	Masculino	Masculino
Edad (años)	43	55	57	53
Cuadro clínico	Dolor, cambio de coloración y edema en la región malar derecha	Cefalea hemicraneana izquierda y disminución de la agudeza visual ipsilateral	Dolor en piezas dentales y disminución de la agudeza visual izquierda	Rinosinusitis crónica purulenta yesión necrótica en el paladar duro
Tiempo de evolución (días)	120	2	5	30
Diabetes mellitus *de novo*	Sí	Sí	No, hace 28 años	No, tiempo desconocido
Consumo de esteroides	No	Sí, 50 mg/día de prednisona desde hace tres años	No	No
HbA1c de ingreso	11,09 %	10,52 %	16,21 %	13,6 %
Sobreinfección bacteriana, al diagnóstico	No	Sí, sin aislamiento microbiológico	Enterobacteriaceae y *Staphylococcus* coagulasa negativo	*Enterobacter cloacae* y *Klebsiella pneumoniae*
Método de diagnóstico	Histopatología	Histopatología y cultivo	Histopatología	Histopatología
Desbridamiento más manejo antimicrobiano	• 5 mg/kg/día de anfotericina B liposómica por 11 días	• 5 mg/kg/día de anfotericina B liposómica por cinco semanas	• 5 mg/kg/día de anfotericina B liposómica por 42 días, luego 10 mg/kg/día durante 24 días ^¶^	• 5 mg/kg/día de anfotericina B liposómica por 22 días
		• Isavuconazol por 14 días	• Caspofungina por 25 días ^¶^	• Isavuconazol indefinido*
		• Posaconazol indefinido*	• Posaconazol indefinido ^¶^*	
Estado al dar de alta	Vivo (alta voluntaria)	Vivo	Vivo	Falleció por bacteriemia causada por *Staphylococcus aureus*

* Tratamiento ambulatorio

^¶^ Combinación de antimicóticos

## Descripción de los casos

### Paciente 1

Se trata de un hombre de 43 años, mestizo, con antecedente de obesidad e historia de cuatro meses de dolor periorbitario derecho, eritema y edema palpebral ipsilateral. Antes de su ingreso hospitalario, recibió aminopenicilinas y cefalosporina por sospecha de celulitis preseptal. El paciente presentó mejoría parcial y recurrencia del cuadro clínico. Se practicó una tomografía computarizada (TC) de senos paranasales que evidenció pansinusitis crónica con destrucción de las paredes óseas del seno maxilar y de la pared lateral de la órbita derecha, además de hallazgos de secuestro óseo. Los exámenes de laboratorio al ingreso revelaron una glucosa de 165 mg/dl y una hemoglobina glicosilada (HbA1c) de 11,09 %.

Con el diagnóstico de diabetes mellitus *de novo* y bajo la sospecha de infección fúngica por el secuestro óseo, se practicó lavado, desbridamiento y toma de muestras del seno maxilar. Mediante examen con hidróxido de potasio, se demostró la presencia de hifas sincitiales (*coenocytic*). La biopsia del maxilar derecho evidenció inflamación granulomatosa con necrosis extensa, y presencia de hifas gruesas y sincitiales (*coenocytic*).

Los hallazgos se interpretaron como mucormicosis y se inició tratamiento con anfotericina B liposómica. El tratamiento de la diabetes mellitus se ajustó con insulina. Luego de completar 12 días de tratamiento efectivo con anfotericina, el paciente firmó su salida voluntaria, a pesar de la intervención realizada por el equipo de psiquiatría.

### Paciente 2

Se trata de un hombre de 55 años, con cefalea hemicraneana frontal izquierda, ptosis palpebral y disminución de la agudeza visual ipsilateral. Tenía antecedentes de asma, sin tratamiento médico dirigido, y se automedicaba, desde hacía tres años, con 50 mg/día de prednisona de forma intermitente y con salbutamol. En la valoración clínica, se encontró facies de luna llena y fragilidad de la piel; además, en el ojo izquierdo, edema palpebral, limitación parcial para todos los movimientos extraoculares, anisocoria y falta de percepción de la luz.

En los exámenes de laboratorio, se documentó HbA1c en 10,52 %. La resonancia magnética mostró trombosis del seno cavernoso del lado izquierdo, engrosamiento de ambos nervios ópticos, proptosis bilateral y pansinusitis. Se tomó una muestra de la lesión, que reveló hifas hialinas sincitiales (*coenocytic*) con ramificaciones de 90° y, en el cultivo, se identificó el hongo *Rhizopus* spp. Se diagnosticó mucormicosis rino-sino-orbital. Se inició tratamiento con insulina y anfotericina B liposómica durante cinco semanas y, posteriormente, posaconazol por tiempo indefinido.

En la cirugía se encontró necrosis bilateral en la mucosa del *septum* nasal y una bola fúngica en el etmoides. El paciente fue sometido a descompresión endoscópica de nervios ópticos, etmoidectomía y esfenoidectomía. Sin embargo, durante su evolución, presentó pérdida de la agudeza visual bilateral, mejoró el compromiso local y continuó el manejo antifúngico de forma ambulatoria.

### Paciente 3

Se trata de un hombre de 57 años con antecedentes de 28 años de diabetes mellitus de tipo 2, con pobre cumplimiento del tratamiento. Consultó por disminución de la agudeza visual en el ojo izquierdo y dolor odontogénico.

**Figura 1:**
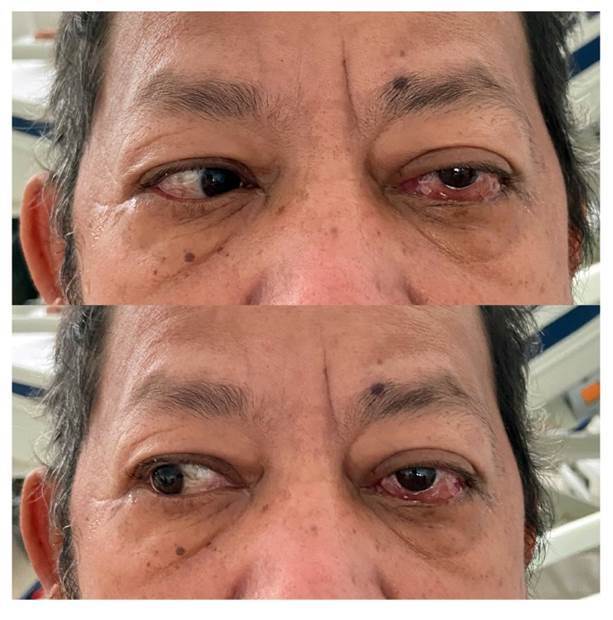
Paciente 3. En el ojo izquierdo, se observa proptosis, oftalmoplejia y edema conjuntival.

El examen físico reveló oftalmoplejia en el ojo izquierdo (figura 1). Se observó una placa blanca purulenta no dolorosa en el paladar. La HbA1c de ingreso fue de 16,21 %. Se sospechó una lesión en el seno cavernoso, y se practicaron una angiotomografía y una resonancia magnética de cabeza, con las que se descartaron lesiones vasculares y se evidenció compromiso intraorbitario izquierdo por edema, con efecto de masa en los tejidos periorbitarios, oclusión de la arteria central de la retina izquierda y ocupación en los senos paranasales con nivel hidroaéreo.

Se practicó una cirugía endoscópica transnasal durante la cual se encontraron áreas necróticas y lesiones de aspecto fúngico en la cavidad nasal, la fosa infratemporal, el piso de la órbita y los senos paranasales. Los estudios histopatológicos reportaron múltiples hifas hialinas sincitiales (*coenocytic*), por lo que se diagnosticó mucormicosis.

Se inició tratamiento con anfotericina B liposómica y se practicó exenteración orbitaria izquierda. Se ajustó el tratamiento de la diabetes mellitus de tipo 2 con insulina con un esquema basal-bolo. A pesar del tratamiento, hubo progresión de la extensión fúngica en la base del cráneo y no fue posible la intervención neuroquirúrgica. Se adicionaron caspofungina y posaconazol al tratamiento. Luego de los múltiples lavados y desbridamientos quirúrgicos, el paciente egresó con posaconazol por término indefinido.

### Paciente 4

Se trata de un hombre de 53 años que consultó por presentar 30 días de evolución de una lesión ulcerada, necrótica, con exposición ósea en el paladar, acompañada de rinorrea y fiebre. Tenía antecedentes de diabetes mellitus de tipo 2, sin tratamiento.

Se tomó una biopsia de la lesión en la que se aislaron estructuras micóticas descritas como hifas hialinas sincitiales (*coenocytic*). Se le diagnosticó mucormicosis, se inició tratamiento con anfotericina B liposómica, y se practicaron desbridamiento y lavado de la lesión. Al ingreso hospitalario, se reportó hemoglobina glicosilada de 13,6 % y una prueba negativa para HIV. Los cultivos mostraron sobreinfección con *Enterobacter cloacae y Klebsiella pneumoniae*. El paciente recibió insulina y antibióticos.

En la resonancia magnética se evidenció compromiso inflamatorio y destrucción ósea de las cavidades paranasales, las celdillas etmoidales, los senos esfenoidales y los huesos maxilares. Había un defecto en el paladar blando y en el duro, y un realce subcutáneo del medio de contraste en la región maxilar perioral e infraorbitaria izquierda. Se procedió a practicar maxilectomía bilateral y etmoidectomía anterior y posterior, con resección del piso de la órbita izquierda y de los cornetes.

Se indicó tratamiento de 22 días con anfotericina B (como terapia de inducción) e isavuconazol de forma indefinida. Sin embargo, pese a la mejoría local de la infección, el paciente falleció por sepsis a causa de una bacteriemia por *Staphylococcus aureus* resistente a la oxacilina.

### Consideraciones éticas

Este trabajo cuenta con el consentimiento informado para la toma y publicación de la fotografía del paciente número 3, después de haberle explicado, claramente y en lenguaje sencillo, las intenciones académicas de la misma, y despejar sus dudas y las de su familiar.

## Discusión

El diagnóstico de la mucormicosis es complejo ya que la infección es causada por más de 25 especies de agentes patógenos. Los métodos de referencia para el diagnóstico son el cultivo y la identificación histopatológica.

En esta serie de casos, el hongo, identificado como *Rhizopus* spp., se aisló en uno de los cuatro pacientes. Los estudios histopatológicos -de coloración básica con hematoxilina y eosina- e histoquímicos, evidenciaron un infiltrado escaso, de tipo inflamatorio mixto, de predominio linfoplasmocitario, y áreas de necrosis y hemorragia.

**Figura 2:**
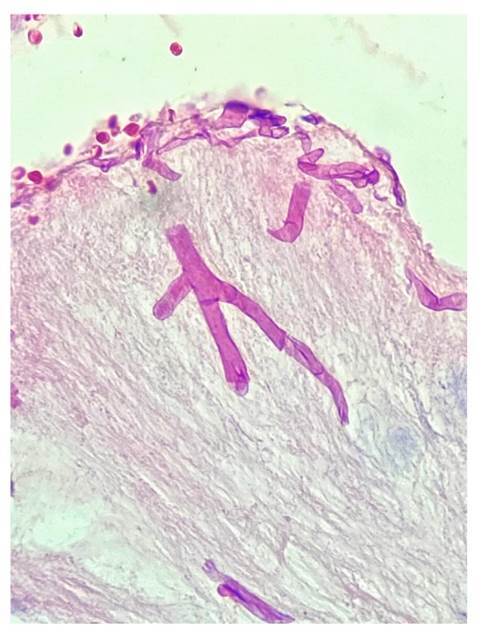
Se observa tejido fibroso con una hifa angulada y ramificada con paredes anchas y sincitiales (*coenocytic*). Hematoxilina y eosina, 40X.

**Figura 3:**
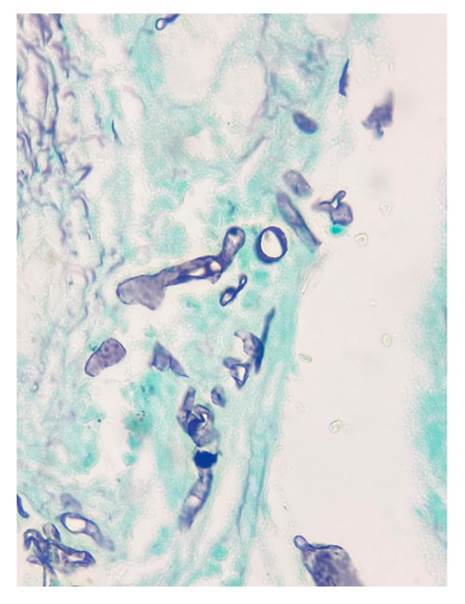
Se reconocen hifas hialinas, gruesas, irregulares, seccionadas de forma transversal y sagital. Gomori-Grocott, 40X.

**Figura 4:**
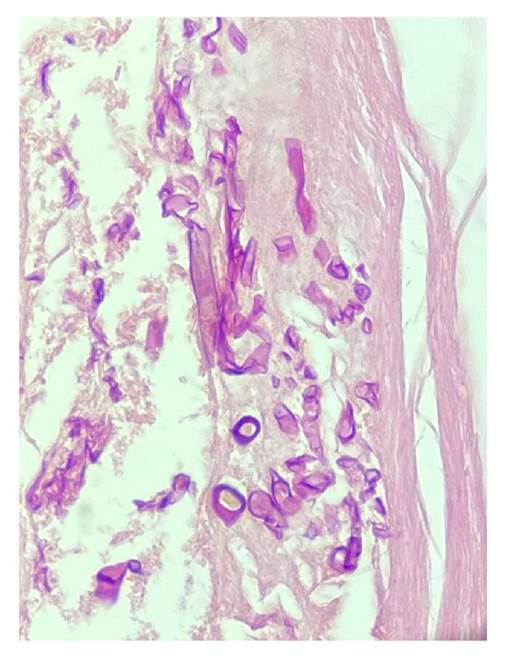
Se observan múltiples estructuras circulares y alargadas de paredes gruesas e irregulares, algunas de ellas sincitiales (*coenocytic*) e incompletas, sobre un estroma densamente fibroso. Hematoxilina y eosina, 40X.

**Figura 5:**
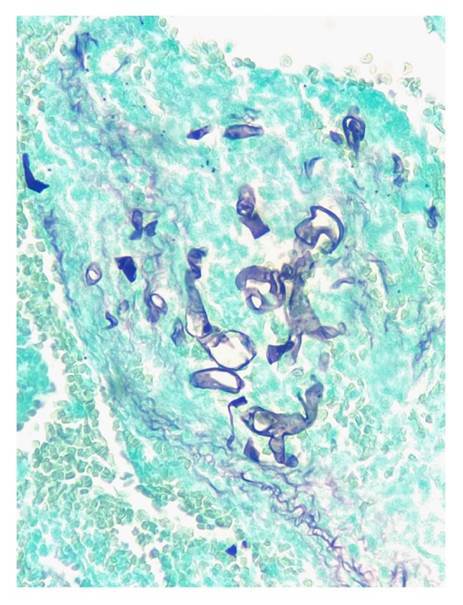
Se identifican hifas hialinas, irregulares, de diferentes formas y paredes gruesas, distribuidas de manera difusa sobre un estroma denso. Gomori-Grocott, 40X.

En todos los casos, se identificaron hifas anchas que variaban entre 5 y 20 pm, de contornos irregulares y pleomorfas. Su patrón de ramificación era al azar y, aunque predominaban en ángulo recto, algunas de ellas estaban colapsadas, organizadas en cordones o con doblamiento (figuras 2-5) ^1^.

En la literatura, el bajo rendimiento de los cultivos se ha relacionado con la recuperación subóptima de los tejidos infectados, la friabilidad de las hifas, la homogenización de la muestra y el crecimiento lento en los medios de cultivo. Sin embargo, los hallazgos histopatológicos obtenidos corresponden con la enfermedad ^2^^,^^4^. El uso de antígenos, como el galactomanano y el β-D-glucano, no es útil en la mucormicosis. También, se pueden usar técnicas moleculares, mediante reacción en cadena de la polimerasa, que detecten fragmentos del ADN del hongo ^1^^,^^3^.

En el momento del ingreso hospitalario, los cuatro pacientes presentaban diabetes mellitus mal controlada, con concentraciones de HbA1c mayores de 10 % (promedio de 12,8 %), y valores de glucemia mayores de 250 mg/dl. Este es el factor de riesgo más frecuentemente asociado con la mucormicosis, ya que contribuye a la disfunción fagocítica que puede favorecer la infección por mucorales. La disfunción de los polimorfonucleares en pacientes diabéticos se caracteriza por una disminución en su quimiotaxis, diapédesis y en la producción de superóxidos ^7^. También, se ha descrito aumento de la expresión de la proteína regulada por glucosa 78 (GRP78) en pacientes diabéticos, un receptor endotelial que favorece la invasión fúngica por mucorales mediante un mecanismo endocítico. La angioinvasión es uno de los mayores factores de virulencia en la mucormicosis ^8^.

El principal mecanismo de defensa del huésped ante la invasión por mucorales, consiste en la reacción inmunológica innata: en primer lugar, los macrófagos tisulares fagocitan las esporas para evitar la formación de hifas. Ante una falla en este mecanismo, los neutrófilos intentan contener la infección mediante la fagocitosis de hifas y el estallido respiratorio ^7^^,^^9^*.* La activación de los receptores reconocedores de patrones, como el TLR-2 en *Rhizopus oryzae*, favorece la producción de citocinas proinflamatorias, predominantemente IL-6 y TNF-a ^8^. Entre otros mecanismos que promueven el crecimiento fúngico de los mucorales, presentes en pacientes diabéticos, se encuentra la hiperglicosilación de proteínas secuestradoras del hierro*,* que causa un aumento del hierro libre en suero e incrementa el crecimiento de hifas ^2^^,^^9^.

Los mecanismos de inmunosupresión descritos en la hiperglucemia persistente incluyen la supresión de la producción de citocinas, los defectos en la fagocitosis y la disfunción de las células del sistema inmunológico ^10^. Uno de los pacientes refirió uso crónico de esteroides, los cuales se asocian con hiperglucemia y alteración del sistema inmunológico ^11^.

*Rhizopus* spp. tiene la enzima cetona reductasa que favorece el crecimiento fúngico en ambientes ácidos y ricos en glucosa, como el de aquellos pacientes con cetoacidosis diabética, aunque cabe resaltar que ninguno de los casos aquí presentados cursó con dicha condición ^11^.

Como se evidencia en los casos expuestos, el tratamiento de la mucormicosis implica una combinación de desbridamiento quirúrgico de los tejidos afectados y tratamiento antifúngico ^12^. En el 100 % de los casos se utilizó el tratamiento quirúrgico. Es necesario el control de los factores que predisponen a la infección, que en todos estos casos fue la hiperglucemia. En el caso número 3, el control glucémico fue difícil y se correlacionó con la progresión de la infección durante la hospitalización, a pesar del tratamiento antimicótico concomitante.

En la serie de casos presentada, se consideró el desbridamiento quirúrgico agresivo de los tejidos involucrados tan pronto como se confirmó el diagnóstico. La intervención quirúrgica con extirpación de tejido necrótico y reducción de la infección, se ha asociado con una mayor supervivencia en las revisiones clínicas sobre infección rinocerebral ^13^.

No existen estudios aleatorios que evalúen la eficacia de los esquemas terapéuticos antimicóticos para la mucormicosis, porque la enfermedad es rara. Sin embargo, la anfotericina B liposómica intravenosa es el fármaco de elección para el tratamiento inicial ^3^^,^^12^. Con la confirmación histológica y en simultáneo con el manejo quirúrgico, se inició el tratamiento con anfotericina B intravenosa en los cuatro pacientes.

El inicio temprano de la terapia antimicótica mejora el desenlace de la infección con mucormicosis ^14^. No se ha estudiado la dosis total de anfotericina B liposómica que debe administrarse; no obstante, la dosis inicial habitual es de 5 mg/kg diarios y, en ocasiones, podría aumentarse hasta 10 mg/kg ^14^. El posaconazol e isoconazol son azoles de amplio espectro, activos *in vitro* contra los agentes de la mucormicosis; se usan como terapia de reducción y rescate para los pacientes que no mejoran o no toleran la anfotericina B ^3^. En este estudio, tres de cuatro pacientes recibieron manejo con azoles, uno de ellos recibió posaconazol en combinación con anfotericina B como terapia de rescate por progresión de la enfermedad.

Con el posaconazol (intravenoso u oral) y para el isoconazol, se deben dar dosis de carga de 300 mg cada 12 horas el primer día, luego una dosis de mantenimiento de 300 mg cada 24 horas y 200 mg intravenoso u oral cada 8 horas durante las primeras seis dosis, seguidas de 200 mg intravenoso u oral cada 24 horas ^3^.

No hay recomendaciones sobre las terapias combinadas y solo hay reportes anecdóticos de combinación de anfotericina B y posaconazol ^15^. En el paciente número 3, se utilizó terapia combinada con caspofungina. Aunque las equinocandinas no tienen actividad *in vitro* contra los agentes de la mucormicosis, existen reportes de que puede tener utilidad clínica, ya que inhibe la enzima 1,3-β-glucano sintetasa expresada por *R. oryzae*. Se evidencia en este caso que, después de la combinación de la triple terapia -anfotericina B intravenosa, posaconazol y caspofungina- presentó estabilización de la infección y continuó solo con azol oral de mantenimiento. El tratamiento debe prolongarse hasta que haya una resolución clínica de los síntomas de la infección y de los signos radiológicos de la enfermedad activa. Con frecuencia, el tratamiento se extiende por meses y algunos pacientes lo reciben de por vida; en tres de los cuatro casos presentados, el tratamiento farmacológico se indicó por tiempo indefinido ^3^^,^^15^.

Además de la terapia combinada, se han utilizado otras estrategias terapéuticas, como el deferasirox -que en ratones ha mejorado la tasa de supervivencia y reducido la carga fúngica en los tejidos- y el oxígeno hiperbárico, sin que se haya establecido completamente su beneficio ^1^^,^^3^^,^^16^.

## Conclusión

Se presenta una serie de cuatro pacientes con diagnóstico de mucormicosis, una entidad infrecuente que afecta a individuos inmunosuprimidos. Todos los pacientes tenían como factor de riesgo diabetes mellitus con inadecuado control metabólico, por lo que es preocupante la predisposición a la infección en casos de diabetes mellitus. Dada la ubicuidad del hongo, es importante controlar los factores de riesgo, como la diabetes, y evitar crisis hiperglucémicas para disminuir la probabilidad de colonización e invasión fúngica. Tres de los pacientes sobrevivieron y todos recibieron tratamiento farmacológico y quirúrgico.

En el tratamiento de la mucormicosis, son fundamentales el control de las comorbilidades, un diagnóstico precoz y una estrategia interdisciplinaria entre los médicos tratantes y el equipo quirúrgico. Existen varios esquemas de tratamiento, pero todos incluyen una inducción con anfotericina B, con opción de dar luego una terapia oral de mantenimiento.

Finalmente, lo que se busca con este trabajo es sensibilizar sobre la enfermedad y que se considere como diagnóstico diferencial en cuadros clínicos rino-cerebro-orbitarios en pacientes con diabetes mellitus.
